# Supplementary material for: INSM1 governs a neuronal progenitor state that drives glioblastoma in a human stem cell model
Source: Nat Commun. 2025 Dec 7;17:31. doi: 10.1038/s41467-025-66371-x (PMC12764576; doi:10.1038/s41467-025-66371-x)
Supplement: Supplementary file 1 — Supplementary Information [file 41467_2025_66371_MOESM1_ESM.pdf]

Supplementary Information

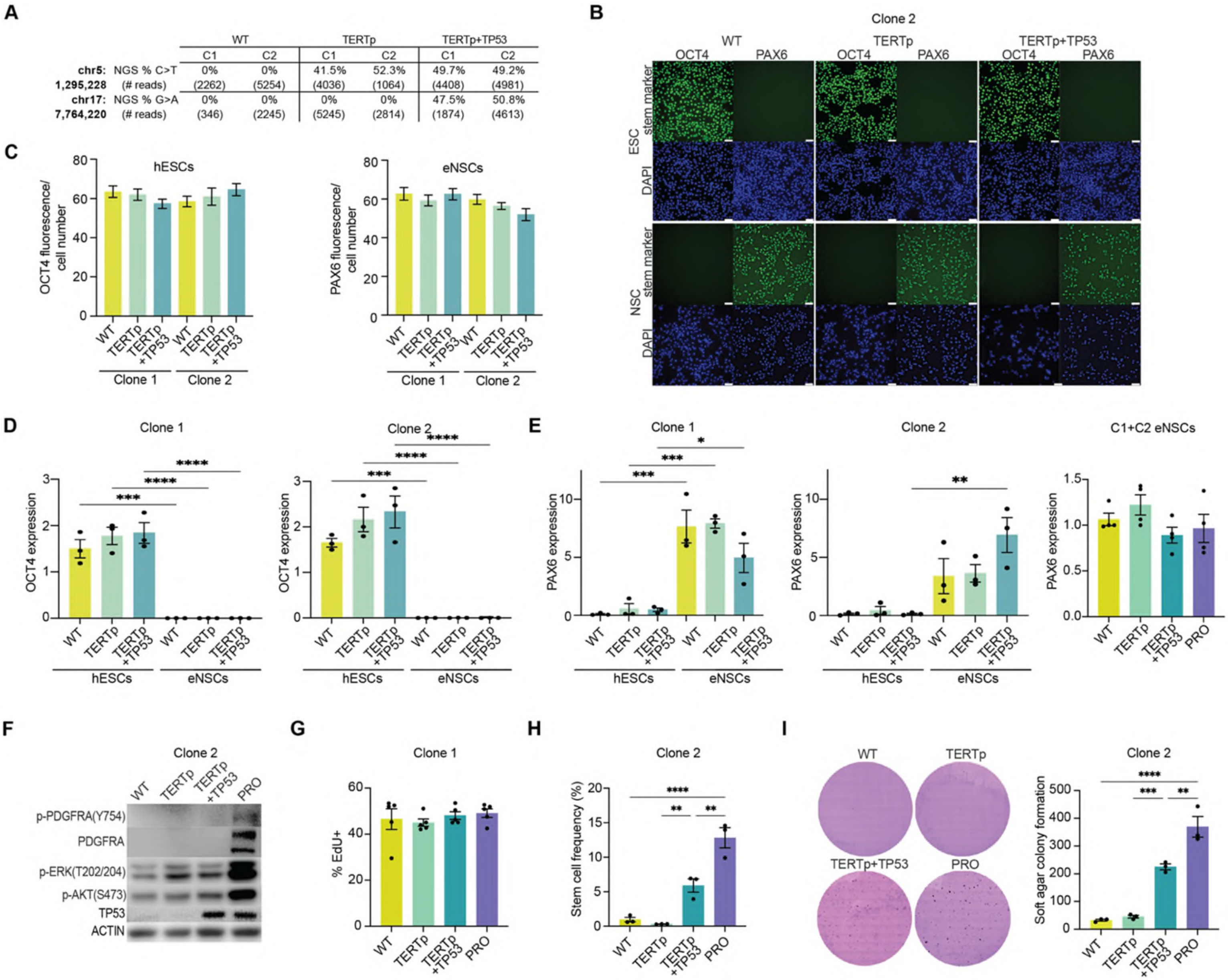

Supplementary Fig. 1: Engineered NSCs (eNSC) display mutant-specific signaling and malignant transformation *in vitro*. A Table of next-generation sequencing (NGS) read counts at *TERT* promoter and *TP53* exon 6 CRISPR-mutated sites in two independent genetic clones (C1 and C2) of wildtype (WT), *TERT*p, and *TERT*p+*TP53* hESCs (clone 1: C1, clone 2: C2). **B** Immunofluorescence (IF) for stem cell markers OCT4 and PAX6 (488 nm), as well as DAPI, in hESC

clone set 2 and their differentiation-matched NSCs (scale bar = 50 μM). Shown is a representative image from 3 independent replicates. **C** Quantification of IF intensity normalized to cell number in independent replicates (n=3, ANOVA, non-significant (ns)). **D** Quantitative RT-PCR for analysis of *OCT4* mRNA expression normalized to *ACTIN* and *GAPDH* expression in independent replicates using two independent genetic clone sets of mutant hESC and matched NSC clones (*n=3*, ANOVA, ***$P<0.001$, ****$P<0.0001$). **E** Quantitative RT-PCR for analysis of *PAX6* mRNA expression normalized to *ACTIN* and *GAPDH* expression in independent replicates using two clone sets of hESC and matched NSC clones (*n*=3 per clone, except on far right where *n=2* per clone in combined plot, ANOVA, *$P<0.05$, **$P<0.01$, ***$P<0.001$). **F** Immunoblot analysis for mutant PDGFRA expression and activity in eNSCs with indicated antibodies. Representative of three independent replicates. **G** Flow cytometric analysis of EdU incorporation for measuring proliferation in eNSC *in vitro* cultures. Data represent mean ± SEM for independent replicates (*n=5*, ANOVA, ns). **H** Extreme limiting dilution assay of serial mutant eNSC genotypes (clone set 2). Data represent mean ± SEM in independent replicates (*n=3*, ANOVA, **$P<0.01$, ****$P<0.0001$). **I** Representative soft agar colony formation assay of eNSCs (left) and quantification (right). Data represent mean ± SEM in independent replicates (*n=3*, ANOVA, **$P<0.01$, ***$P<0.001$, ****$P<0.0001$).

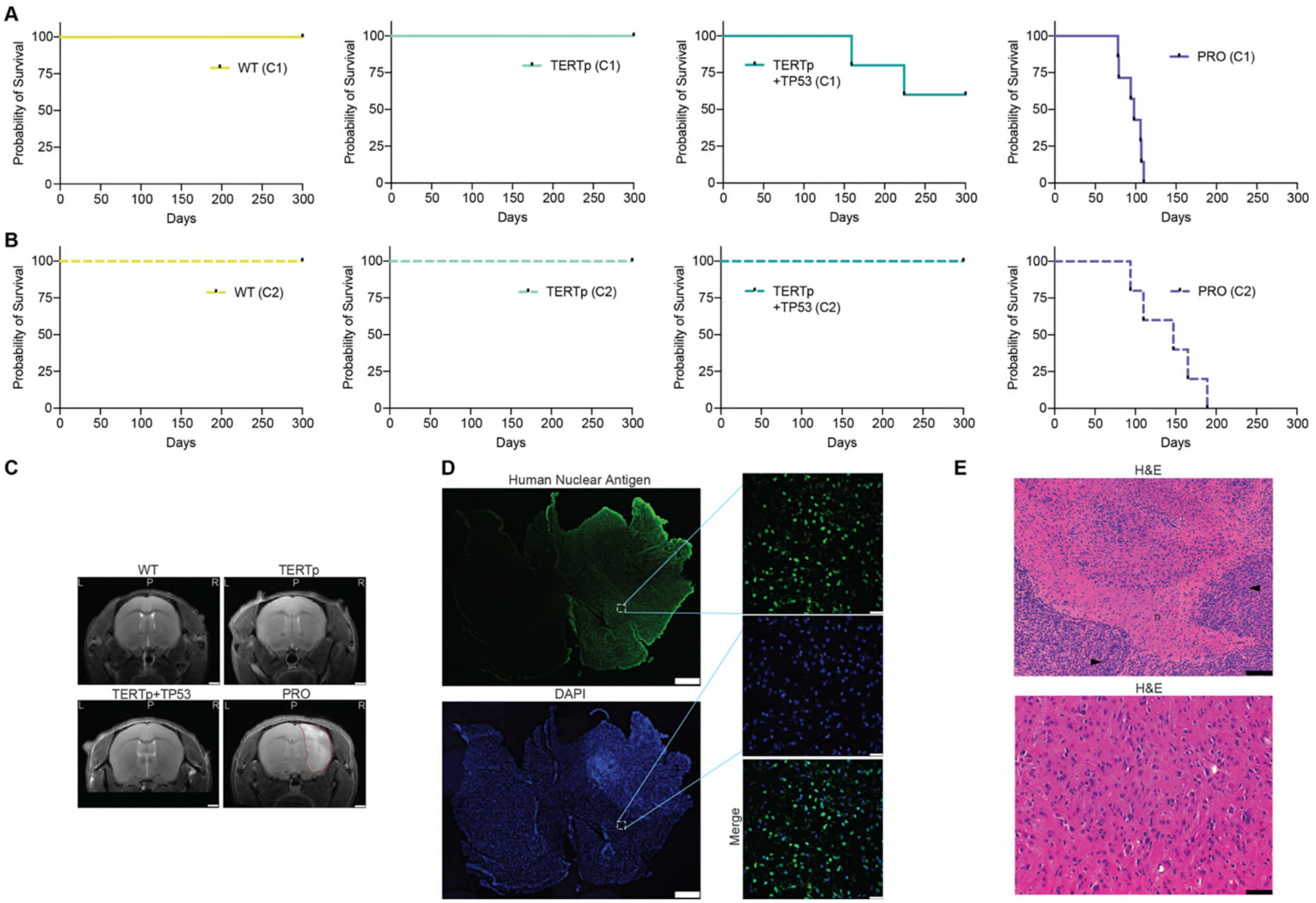

Supplementary Fig. 2: PRO eNSCs form tumors in mice that recapitulate GBM. A Kaplan-Meier survival curves for mice orthotopically injected with eNSCs of each serial mutant genotype (clone set 1). **B** Kaplan-Meier survival curves for mice injected with clone 2 of each genotype. **C** Representative T2-weighted MRI images of eNSC-xenografted mice from each experimental group using serial mutant PRO eNSC genotypes (clone set 2). Red outlines indicate pathological lesions in the coronal plane at injection site (scale bar = 1 mm; L = left, P = posterior, R = right). **D** Immunofluorescence imaging of a representative PRO eNSC brain tumor (clone 2). Frozen sections were stained for Human Nuclear Antigen (488 nm) and DAPI (scale bar = 0.5 mm). Inset images display magnified fields of view and merged images (scale bar = 50 µm). **E** H&E staining of a representative PRO eNSC brain

tumor (clone 2) demonstrates characteristic histopathology of GBM. Pink necrotic center (n) surrounded by pseudopalisading tumor cells (arrowheads), (top, scale bar = 200 μm). Tumor hypercellularity and mitotic atypia (bottom, scale bar = 50 μm).

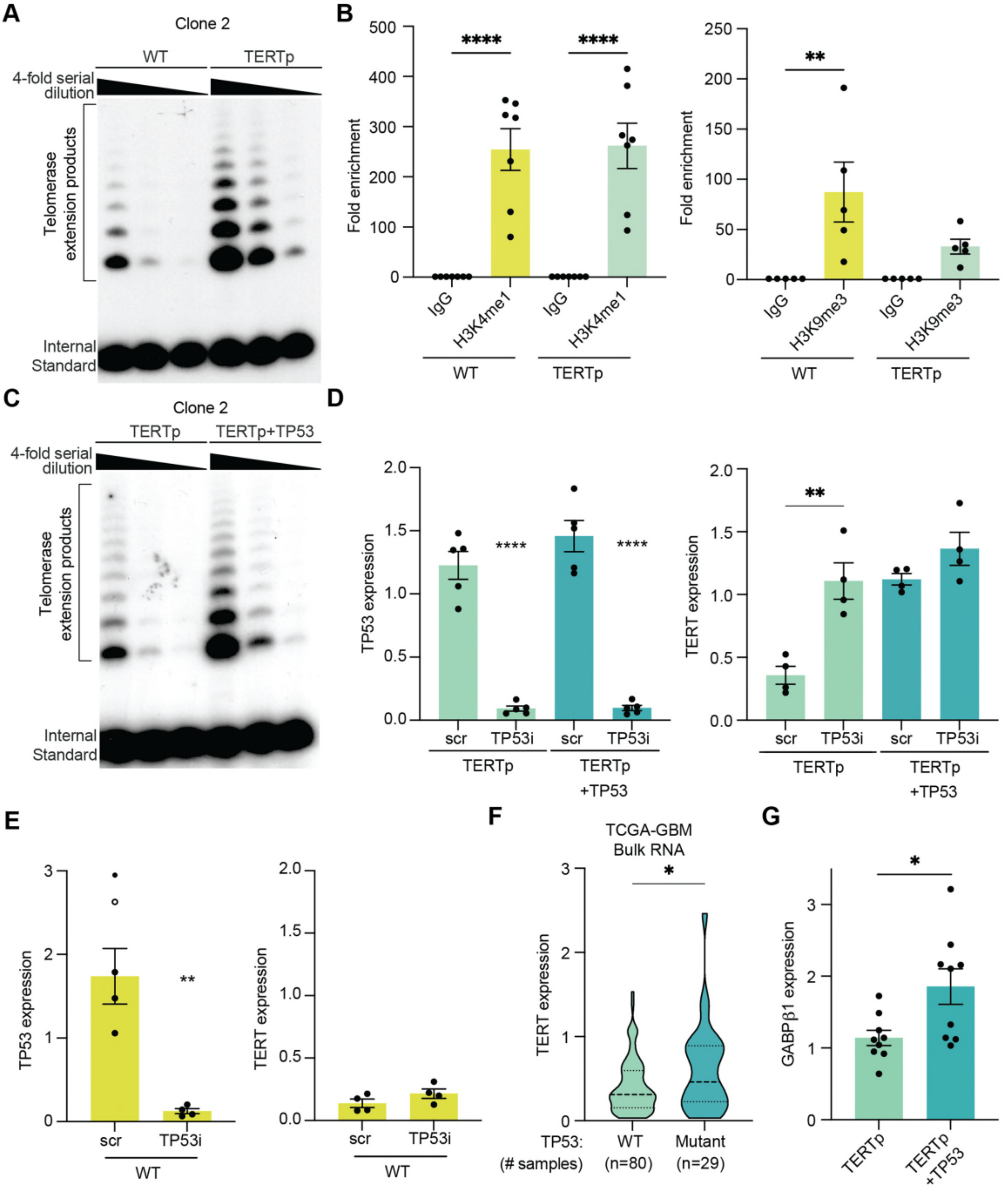

A
Clone 2
WT
TERTp
4-fold serial dilution
Telomerase extension products
Internal Standard
B
Fold enrichment
****
****
**
IgG
H3K4me1
H3K9me3
WT
TERTp
C
Clone 2
TERTp
TERTp+TP53
4-fold serial dilution
Telomerase extension products
Internal Standard
D
TP53 expression
TERT expression
****
****
**
scr
TP53i
TERTp
TERTp +TP53
E
TP53 expression
TERT expression
**
scr
TP53i
WT
F
TCGA-GBM Bulk RNA
TERT expression
*
TP53:
(# samples)
WT (n=80)
Mutant (n=29)
G
GABPβ1 expression
*
TERTp
TERTp +TP53

Supplementary Fig. 3: *TP53* loss-of-function increases TERT expression in GBM. A Telomere Repeat Amplification Protocol (TRAP) Assay for WT and T*ERT*p mutant eNSCs to measure endogenous TERT protein activity. Samples were analyzed with two 4-fold serial dilutions along with an internal standard for amplification process control. The molecular ladder indicates the number of telomeric repeats added to substrate. Data are representative of 3 independent replicates. **B** CUT&RUN coupled with quantitative RT-PCR (qRT-PCR) analysis of H3K4me1 (left) and H3K9me3 levels (right) at the *TERT* promoter in WT and *TERT*p eNSCs. Data represent mean ± SEM in combined clones 1 and 2 in independently replicates and normalized to IgG (=1) (H3K4me1: *n=7*, H3K9me3: *n=5*, ANOVA, $**P<0.01$). **C** TRAP assay of *TERT*p and *TERT*p+*TP53* mutant eNSCs. Assays were performed as in **A**. Data are representative of 3 independent replicates. **D** Quantitative RT-PCR measuring *TP53* (left) and *TERT* mRNA expression (right) normalized to *ACTIN* and *GAPDH* expression following *TP53* RNAi in *TERT*p and *TERT*p+*TP53* eNSCs. Data represent mean ± SEM combining independent genetic clones (clones 1+2) in independent replicates (*n=4*, ANOVA, $**P<0.01$, $****P<0.0001$). **E** Quantitative RT-PCR measuring *TP53* (left) and *TERT* expression (right) normalized to *ACTIN* and *GAPDH* expression following *TP53* RNAi in WT eNSCs. Data represent mean ± SEM combining independent genetic clones (clones 1+2) in independent replicates (*n=4*, ANOVA, $**P<0.01$). **F** Analysis of *TERT* mRNA expression in TCGA-GBM bulk RNA data where samples are stratified by *TP53* mutation status from matched whole exome data (Student's t test, two-sided, $*P<0.05$). **G** *GABPβ1* expression by qRT-PCR normalized to *ACTIN* and *GAPDH* expression in *TERT*p and *TERT*p+TP53 eNSCs. Data represent mean ± SEM combining independent genetic clones (clones 1+2) in independent replicates (*n=8*, Student's t test, two-tailed, $*P<0.05$)

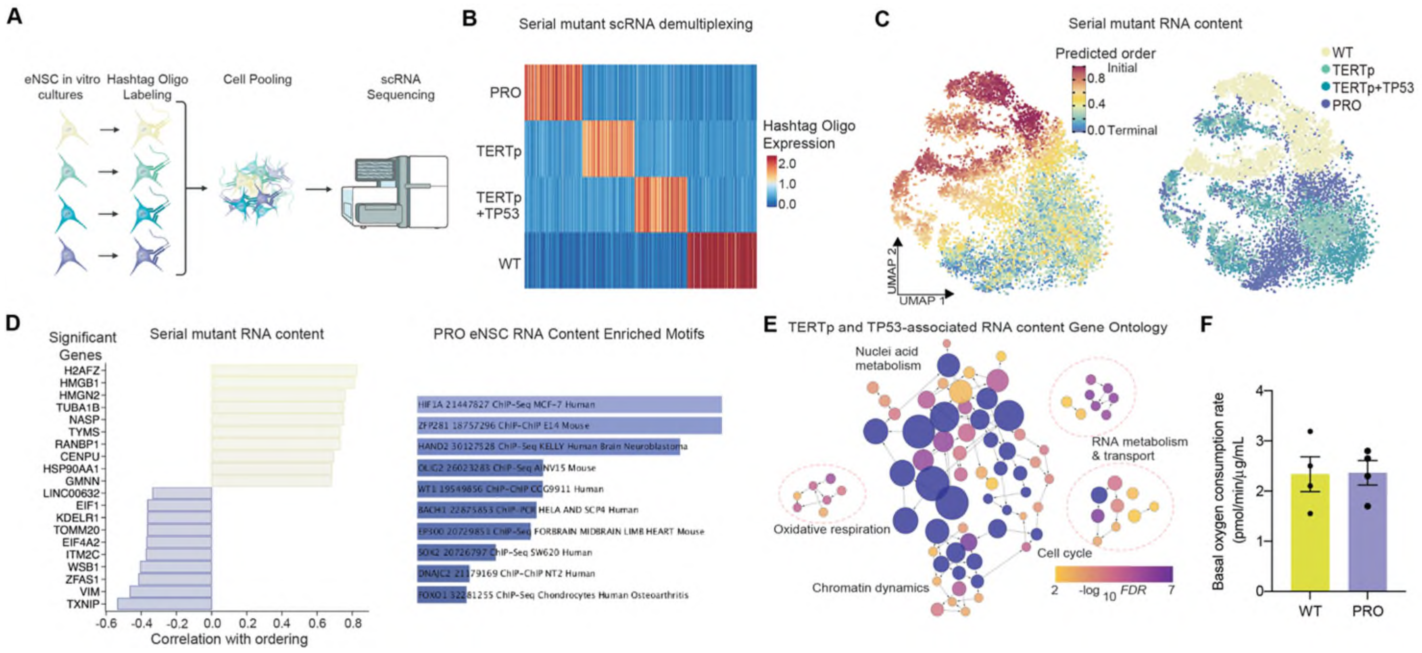

Supplementary Fig. 4: Hashtag oligos allow multiplexed scRNA-seq for trajectory analysis of mutant eNSCs. **A** Schematic of hashtag labeling strategy and pooling for scRNA-seq. **B** Heatmap of demultiplexed hashtag oligo expression by eNSC genotype. **C** UMAP reduction guided by RNA content and colored by predicted ordering (left) and genotype (right). **D** Top 10 genes most highly correlated with initial and terminal ordering by RNA content (left) and motif enrichment analysis in genes associated with terminal ordering (right). **E** Gene ontology of signature representing the middle 50% of inferred ordering associated with *TERT*p and *TERT*p+TP53 eNSCs (n=1,141). Circle size represents number of genes within each ontology, and color indicates log-transformed FDR. **F** Seahorse XF analysis of mitochondria-dependent basal oxygen consumption rate in WT and PRO eNSCs. Data represent mean ± SEM in independent replicates (*n=4*, ANOVA, ns).

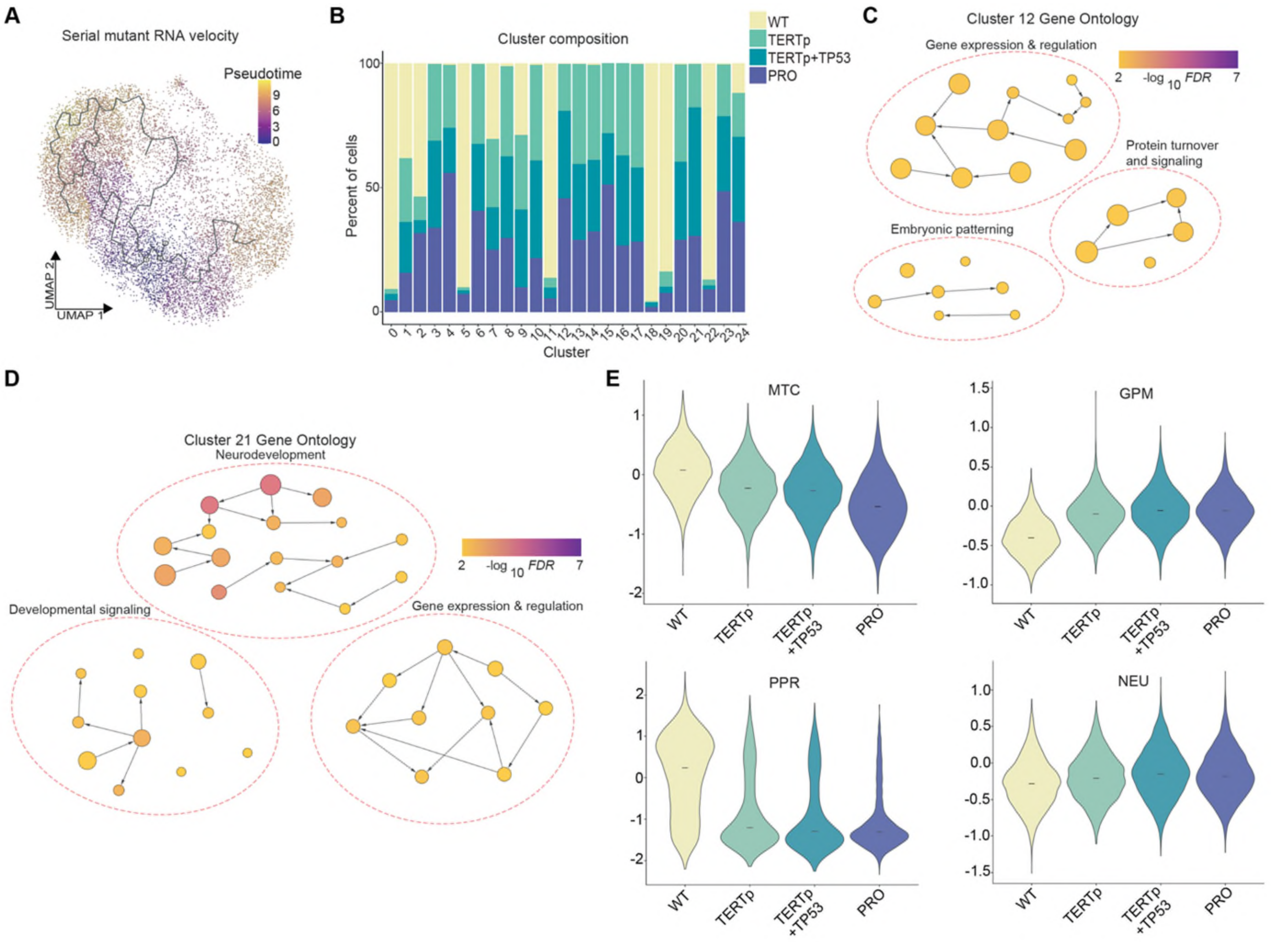

Supplementary Fig. 5: RNA velocity uncovers enrichment of neuronal lineage phenotypes during serial mutagenesis *in vitro*. **A** Pseudotime analysis of serial mutant eNSCs using RNA velocity-based UMAP embeddings. **B** Proportions of each genotype within clusters defined by RNA velocity. **C** Gene ontology analysis of cluster 12-specific and **D** cluster 21-specific genes. Circle size represents number of genes within each ontology, and color indicates log-transformed FDR. **E** Violin plots of *in vitro* serial mutant eNSCs scored for expression of genes defining GBM functional cell states. Shown are the metabolism-related mitochondrial (MTC) and glycolytic/plurimetabolic (GPM) subtypes, as well as the development-related proliferating progenitors (PPR) and neuronal (NEU) subtypes across *in vitro* serial mutants. Horizontal lines within each state indicate median subtype scores.

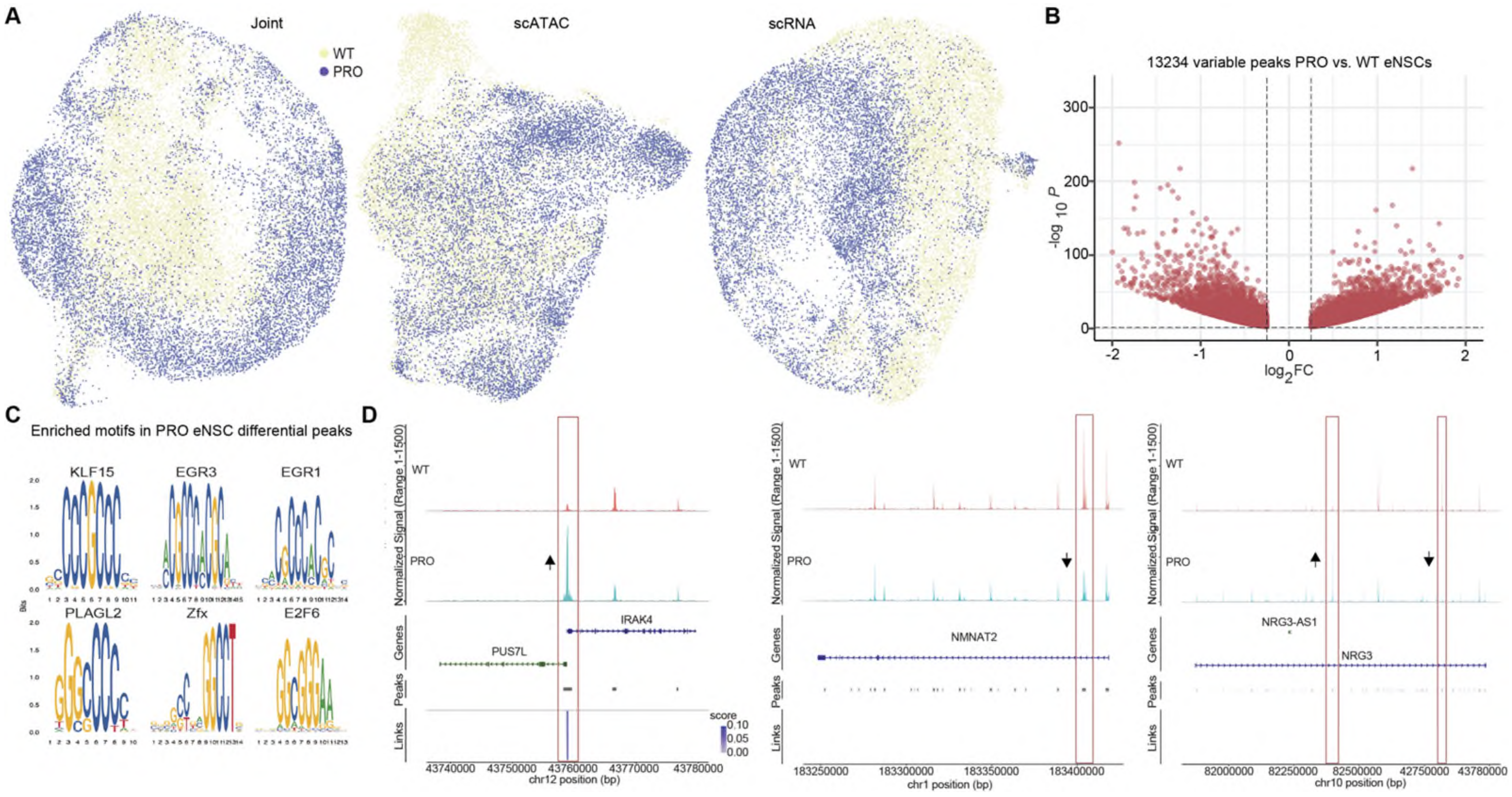

Supplementary Fig. 6: Multiomic analysis of gene expression and chromatin accessibility in single cells of WT and PRO eNSCs dissects transcription factor networks in variable peaks. A UMAP reduction of joint scRNA+scATAC (left), scATAC only (middle), and scRNA only (right) data from WT and PRO eNSC multiomics. **B** Variable peaks in PRO eNSCs compared to WT NSCs. **C** Transcription factor motifs enriched in PRO eNSC variable peaks**.** **D** Examples of differentially accessible peaks in *IRAK4/PUS7L* (left), *NMNAT2* (middle) and *NRG3* genes (right). Red boxes highlight variable peaks at these loci, and arrows indicate direction of peak change in PRO eNSCs vs. WT NSCs.

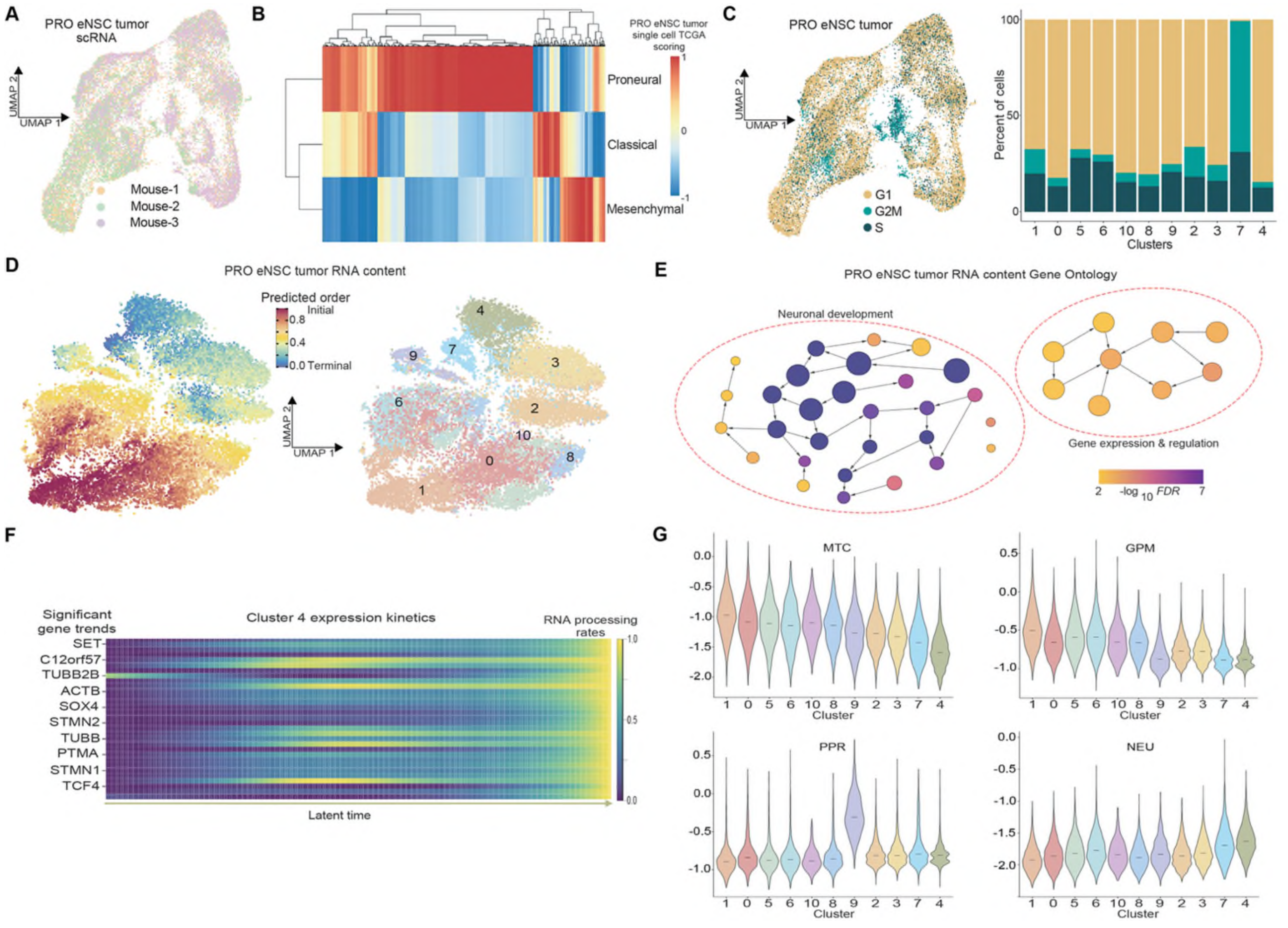

Supplementary Fig. 7: PRO eNSC tumors exhibit metabolic to developmental gene expression changes during evolution. **A** PCA-based UMAP of PRO eNSC tumor cells collected from 3 mice. **B** Single cell classification of PRO eNSC tumors by TCGA subtype gene set enrichment scoring. **C** Cell cycle analysis of PRO eNSC tumors showing UMAP colored by cell cycle (left) and composition of tumor clusters by cell cycle phase proportions (right). **D** UMAP reductions constrained by RNA content in PRO eNSC tumors and colored by predicted ordering (left) and cluster (right). **E** Gene ontology analysis of genes most highly associated with the terminal cluster 4 subpopulation. Circle size represents the number of genes within each ontology, and color indicates log-transformed FDR. **F** Analysis of significant gene expression trends in cluster 4 using RNA processing rates and scaled expression over latent time. Transcript initiation rate, splicing rate, degradation rate are dynamically

modeled to determine lineage-defining gene expression changes. **G** Violin plots of PRO eNSC tumors cell clusters scored for expression of genes defining GBM functional cell states. Shown are the MTC (mitochondrial), GPM (glycolytic/plurimetabolic), PPR (proliferating progenitor), and NEU (neuronal) subtypes across PRO eNSC tumor clusters. Horizontal lines within each state indicate median subtype scores.

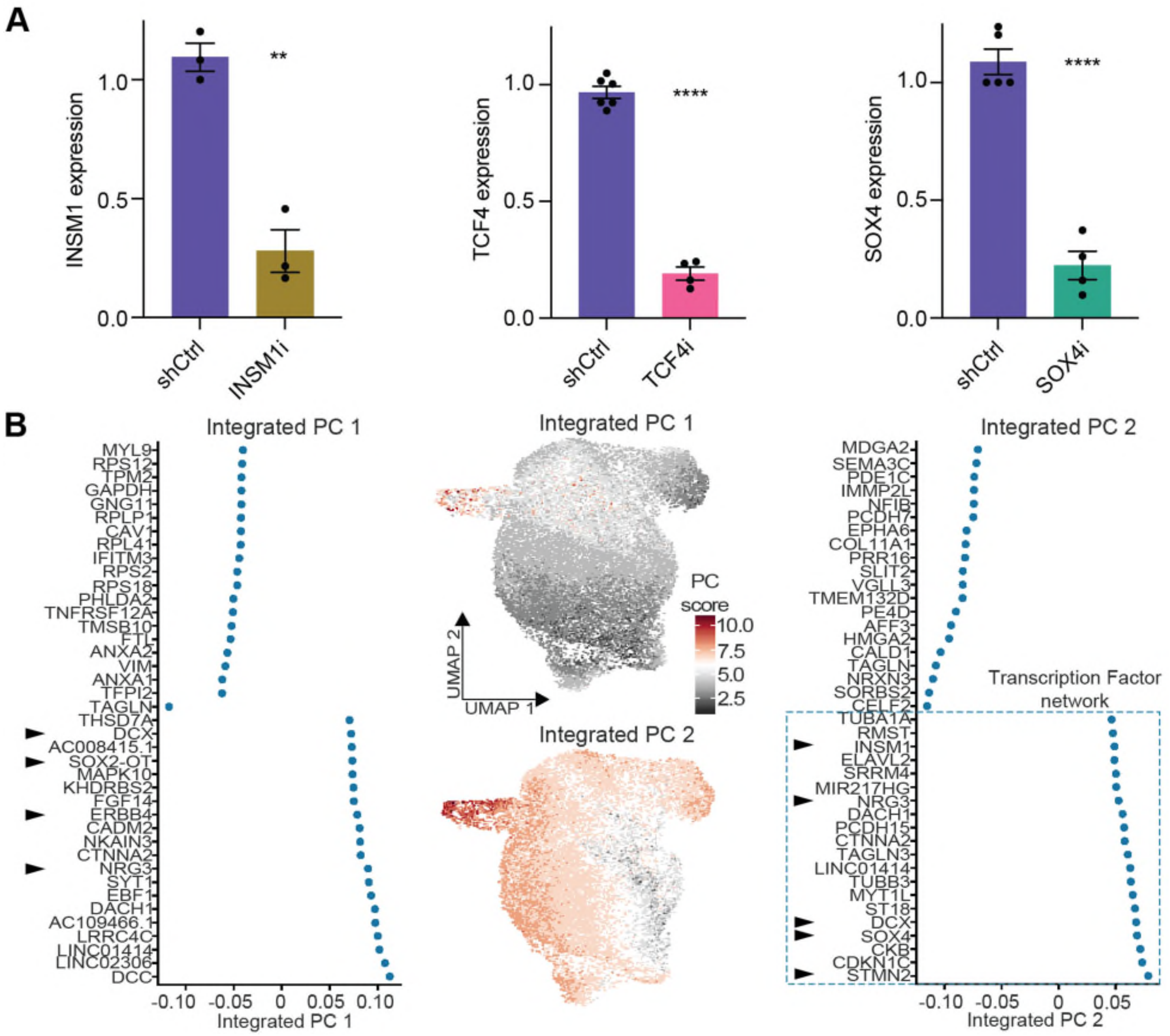

Supplementary Fig. 8: Inhibiting transcriptional drivers of PRO eNSC evolution disrupts convergent gene expression networks. A Expression of *INSM1*, *TCF4*, or *SOX4* mRNA by qRT-PCR normalized to *ACTIN* and *GAPDH* expression in PRO eNSCs (clone 1) following inhibition using a second, independent RNAi targeting indicated genes (vs. shCtrl). Data represent mean ± SEM in independent replicates (INSM1i: $n=3$; TCF4 and SOX4i: $n=4$, Student's t test, $^{**}P<0.01$, $^{****}P<0.0001$). **B** First (left and top middle) and second (right and bottom middle) principal components of the PRO eNSC integrated RNAi scRNA dataset highlighting the variation captured in the dimension-reduced dataset (middle panels) and the genes most highly correlated (PC>0) or anti-correlated

(PC<0) with the variation (left and right panels). Arrowheads indicate genes relevant to PRO eNSC evolution *in vitro* or *in vivo*. The transcription factor network perturbation upon candidate gene inhibition is highly correlated with the second principal component.

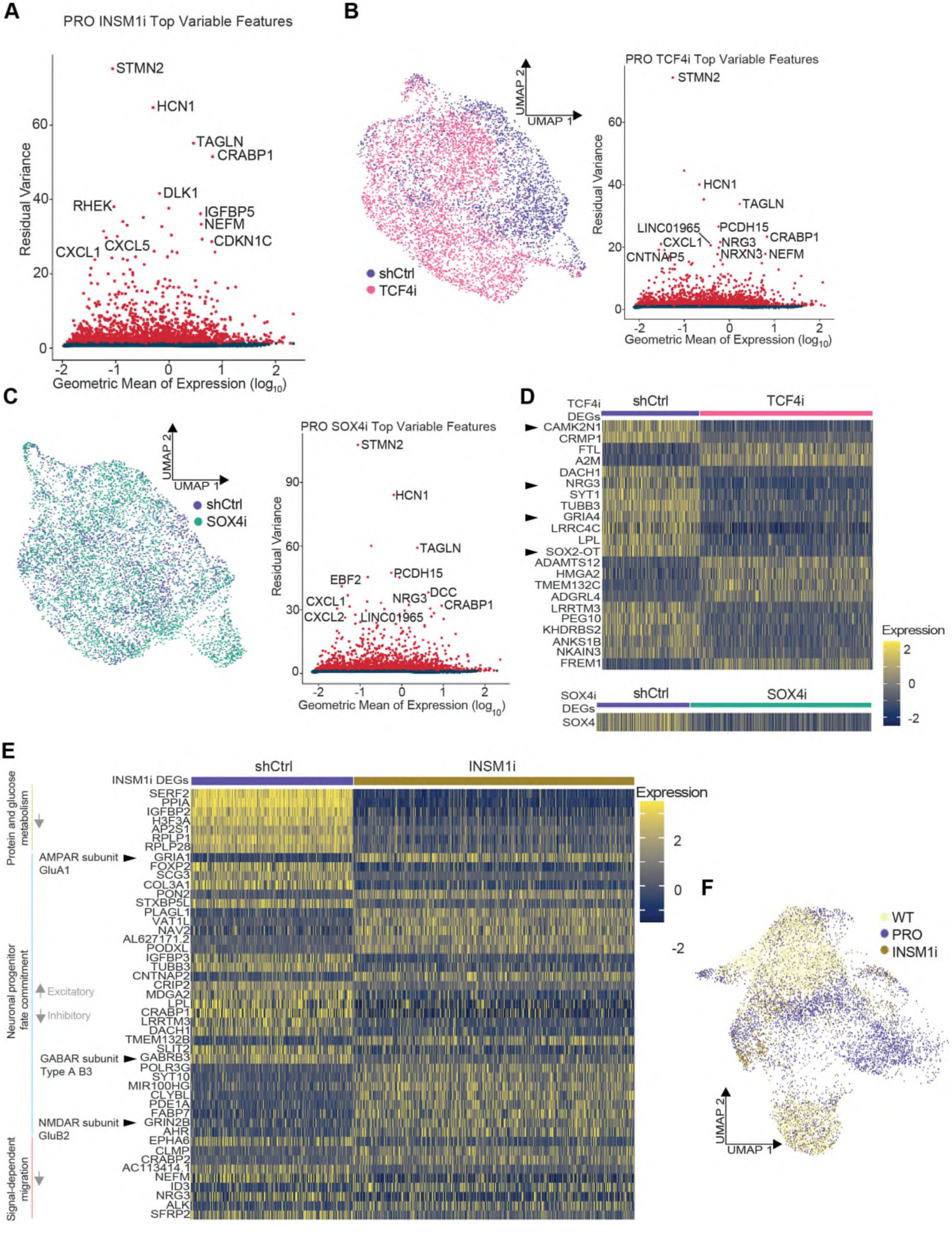

A
PRO INSM1i Top Variable Features
STMN2
HCN1
TAGLN
CRABP1
DLK1
RHEK
IGFBP5
NEFM
CDKN1C
CXCL1
CXCL5
Residual Variance
Geometric Mean of Expression (log10)
B
shCtrl
TCF4i
UMAP 2
UMAP 1
PRO TCF4i Top Variable Features
STMN2
HCN1
TAGLN
LINC01965
PCDH15
CXCL1
NRG3
CRABP1
CNTNAP5
NRXN3
NEFM
C
shCtrl
SOX4i
PRO SOX4i Top Variable Features
STMN2
HCN1
TAGLN
EBF2
PCDH15
NRG3
DCC
CXCL1
CRABP1
CXCL2
LINC01965
D
TCF4i DEGs
shCtrl
TCF4i
CAMK2N1
CRMP1
FTL
A2M
DACH1
NRG3
SYT1
TUBB3
GRIA4
LRRC4C
LPL
SOX2-OT
ADAMTS12
HMGA2
TMEM132C
ADGRL4
LRRTM3
PEG10
KHDRBS2
ANKS1B
NKAIN3
FREM1
SOX4i DEGs
SOX4i
SOX4
Expression
E
INSM1i DEGs
shCtrl
INSM1i
Protein and glucose metabolism
SERF2
PPIA
IGFBP2
H3F3A
AP2S1
RPLP1
RPLP28
AMPAR subunit GluA1
GRIA1
FOXP2
SCG3
COL3A1
PON2
STXBP5L
PLAGL1
VAT1L
NAV2
AL627171.2
PODXL
IGFBP3
TUBB3
CNTNAP2
CRIP2
MDGA2
LPL
CRABP1
LRRTM3
DACH1
TMEM132B
SLIT2
GABRB3
POLR3G
SYT10
MIR100HG
CLYBL
PDE1A
FABP7
GRIN2B
AHR
EPHA6
CLMP
CRABP2
AC113414.1
NEFM
ID3
NRG3
ALK
SFRP2
Neuronal progenitor fate commitment
Excitatory
Inhibitory
GABAR subunit Type A B3
NMDAR subunit GluB2
Signal-dependent migration
Expression
F
WT
PRO
INSM1i

Supplementary Fig. 9: Inhibiting transcriptional drivers of PRO eNSC evolution disrupts the oncogenic gene expression landscape. A Top variable features in PRO INSM1i dataset. **B** PRO TCF4i PCA-based UMAP (left) and top 10 variable features in PRO eNSC TCF4i scRNA dataset (right). **C** PRO SOX4i PCA-based UMAP (left) and top 10 variable features in PRO eNSC SOX4i scRNA dataset (right). **D** Top differentially expressed genes following TCF4i in PRO eNSCs (top) and statistically differentially expressed genes following SOX4i in PRO eNSCs (bottom). **E** Top differentially expressed genes following INSM1i in PRO eNSCs. Genes grouped by Reactome pathway analysis. Gray arrows indicate direction of pathway change following INSM1i (vs. shCtrl). Black arrowheads indicate key neuronal ion channel genes defining excitatory and inhibitory fate commitment changes upon INSM1i. **F** Integrated PCA-based UMAP of WT, PRO, and INSM1i eNSCs.

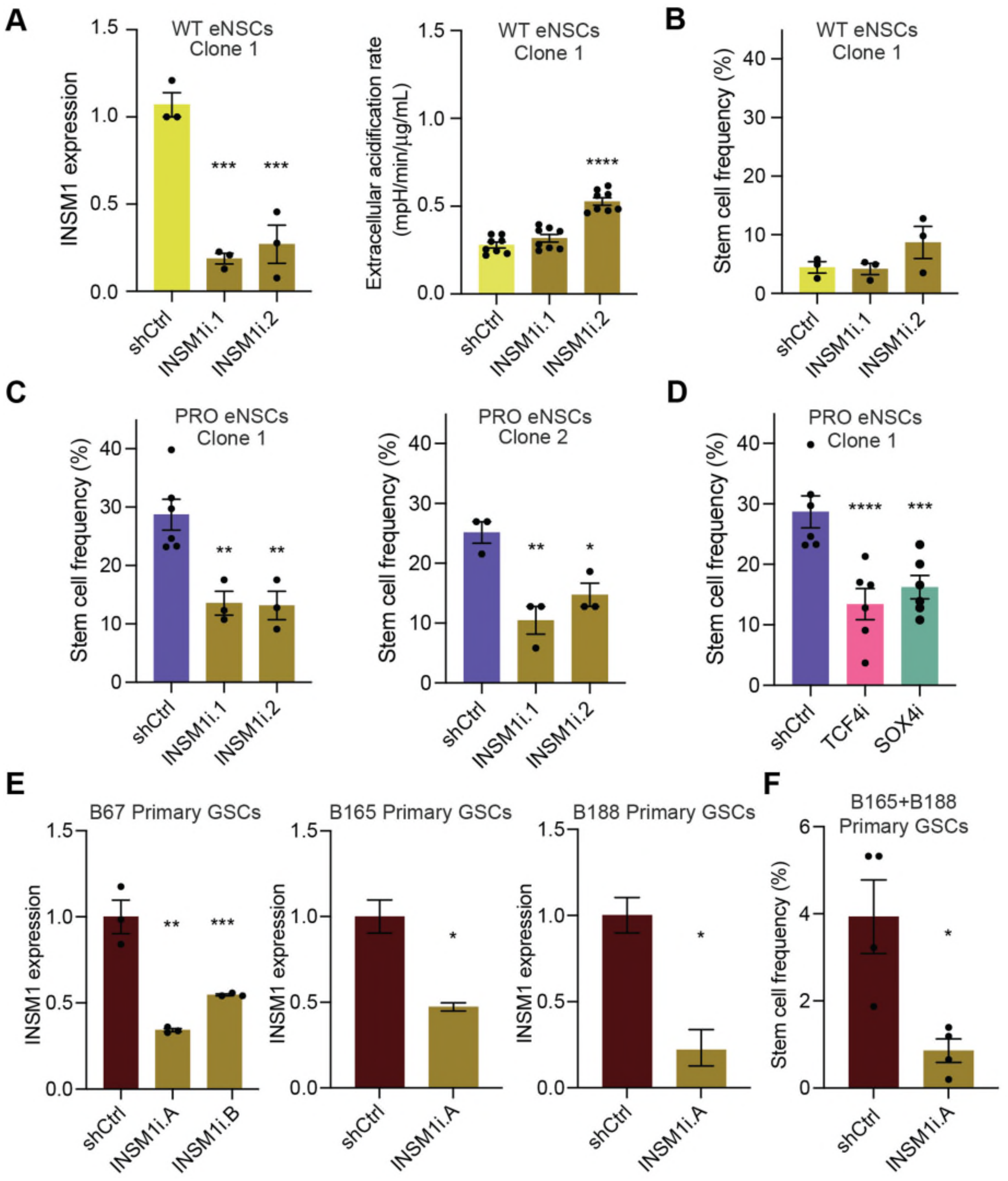

Supplementary Fig. 10: Inhibiting transcriptional drivers of PRO eNSC evolution disrupts self-renewal capacity and stress-related glycolytic pathways. A Left: Expression of INSM1 mRNA by qRT-PCR normalized to ACTIN and GAPDH expression in WT eNSCs (clone 1) following *INSM1* RNAi. Data represent mean ± SEM in independent (*n=3*, ANOVA, ***P*<0.01). Right: Seahorse XF

analysis of glycolysis-dependent extracellular acidification rate in WT eNSCs (clone 1) following *INSM1* RNAi. A slight increase in glycolytic activity was observed with only one *INSM1* RNAi. Data represent mean ± SEM in independent replicates. (*n*=8, ANOVA, ****P<0.0001). **B** Extreme limiting dilution assay using WT eNSCs (clone 1) following *INSM1* RNAi. Data represent mean ± SEM in independent replicates (*n=3*, ANOVA, ns). **C** Self-renewal capacity was measured by extreme limiting dilution assay using PRO eNSCs clone 1 (left) and clone 2 (right) following *INSM1* RNAi using two independent RNAi hairpins. Data represent mean ± SEM in independent (*n=3*, ANOVA, *P<0.05, **P<0.01). **D** Extreme limiting dilution assay using PRO eNSCs (clone 1) following *TCF4 or SOX4* RNAi using two independent RNAi hairpins (Two RNAi per each targeted gene combined in plot). Data represent mean ± SEM in independent (*n=3* per RNAi, ANOVA, ***P<0.001, ****P<0.0001). **E** INSM1 mRNA expression by qRT-PCR normalized to ACTIN and GAPDH expression in B67 (left), B165 (middle), and B188 (right) primary human GBM stem-like cells (GSCs) following *INSM1* RNAi (GIPZ RNAi, Methods). Data represent mean ± SEM in independent replicates (B67, B165: *n=3*; B188: *n=2*, ANOVA, **P<0.01, ***P<0.001). **F** Extreme limiting dilution assay in combined B165 and B188 primary GSCs following inhibition of *INSM1* using RNAi. Data represent mean ± SEM in independent replicates (B165: *n=2*; B188: *n=2*, Student's t-test, two-tailed, *P<0.05).

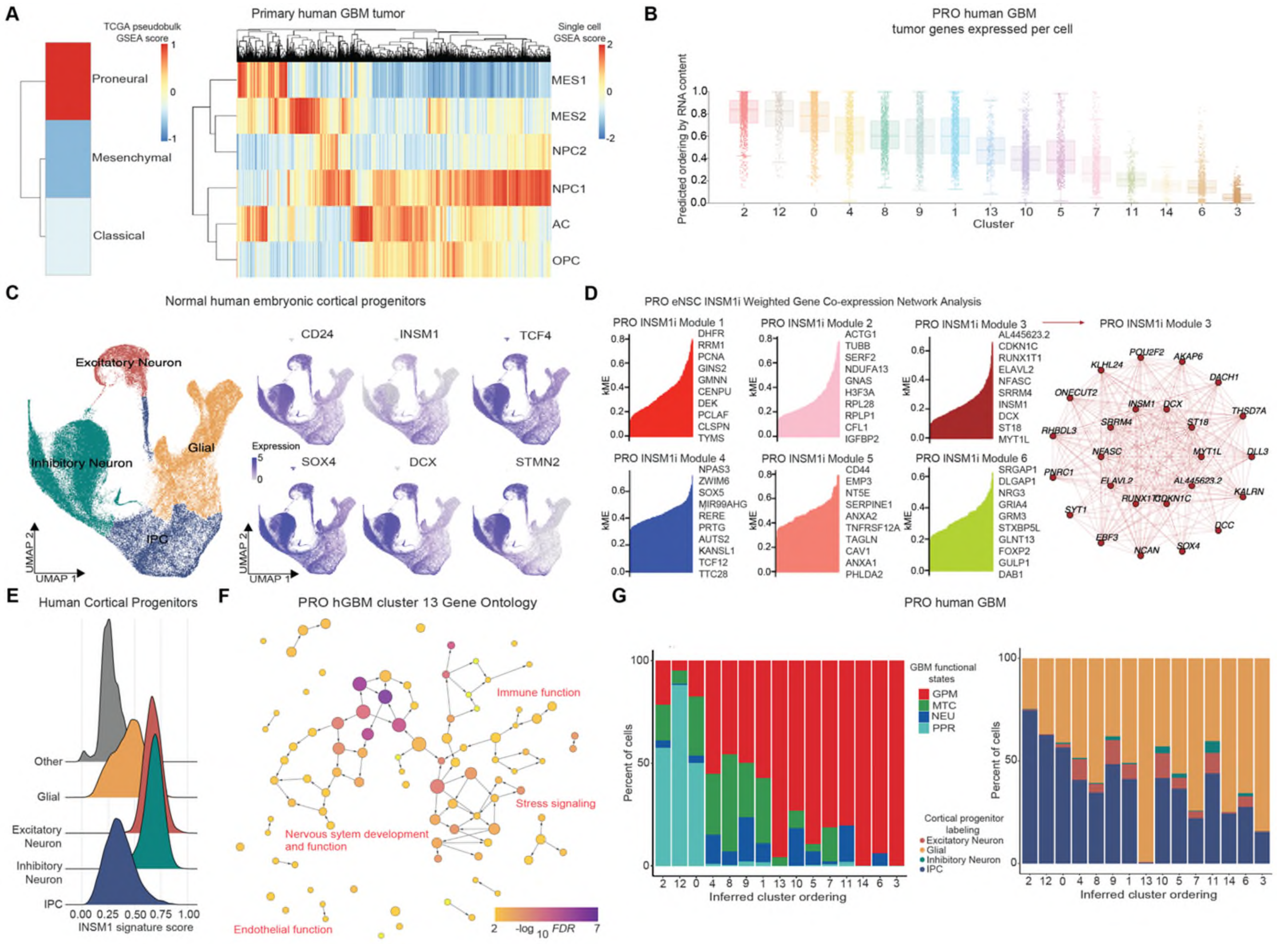

Supplemental Fig. 11: The INSM1 program in PRO GBM reprograms intermediate neuronal progenitor networks. A Pseudobulk enrichment analysis of human GBM tumor using TCGA GBM subtype gene sets (left). Single cell enrichment analysis of human GBM tumor by cell state gene sets defined in Neftel *et al.* 2019 (right). **B** Trajectory inference of PRO human GBM tumor cells by RNA abundance in scRNA-seq dataset. Cluster identities determined by RNA velocity-based UMAP. Box plots indicate the median (center line), interquartile range (hinges), and 1.5x interquartile range (whiskers). **C** Atlas of human cortical progenitor cells from Delgado *et al*. 2022. UMAP is shown (left). Expression of PRO eNSC key INSM1-related genes in transcriptomic

landscape of normal human cortical progenitors (right). **D** High-dimensional Weighted Gene Co-expression Analysis of PRO eNSC INSM1i scRNA-seq data. Shown are module eigengenes, which are calculated by performing PCA on the subset of genes defining each co-expression module. Y-axis represents eigengene-based connectivity, kME, a measure of pairwise correlations between variation in module eigengenes and all genes. Red arrow highlights expanded module 3 eigengene co-expression network hub. **E** INSM1 signature scores in normal human embryonic cortical progenitor cells types. **F** Gene ontology analysis of cluster 13-specific genes. Circle size represents number of genes within each ontology, and color indicates log-transformed FDR. **G** Proportions of GBM functional cell state labels within each cluster of PRO human GBM cell in the scRNA-seq dataset determined by gene set enrichment analysis (left). Proportions of cortical progenitor cell type labels within each cluster of PRO human GBM tumor cells. Clusters ordered by RNA content inference (right).
