# Supplementary material for: INSM1 governs a neuronal progenitor state that drives glioblastoma in a human stem cell model
Source: Nat Commun. 2025 Dec 7;17:31. doi: 10.1038/s41467-025-66371-x (PMC12764576; doi:10.1038/s41467-025-66371-x)
Supplement: Supplementary file 2 — Description of Additional Supplementary Files [file 41467_2025_66371_MOESM2_ESM.pdf]

Title: Supplementary Data 1

Description: INSM1 signature gene list
