# Supplementary material for: INSM1 governs a neuronal progenitor state that drives glioblastoma in a human stem cell model
Source: Nat Commun. 2025 Dec 7;17:31. doi: 10.1038/s41467-025-66371-x (PMC12764576; doi:10.1038/s41467-025-66371-x)
Supplement: Supplementary file 4 — Reporting Summary [file 41467_2025_66371_MOESM4_ESM.pdf]

nature portfolio

Corresponding author(s): Albert H. Kim

Last updated by author(s): 09/13/2025

Reporting Summary

Nature Portfolio wishes to improve the reproducibility of the work that we publish. This form provides structure for consistency and transparency in reporting. For further information on Nature Portfolio policies, see our Editorial Policies and the Editorial Policy Checklist.

Statistics

For all statistical analyses, confirm that the following items are present in the figure legend, table legend, main text, or Methods section.

n/a	Confirmed	
☐	☒	The exact sample size (*n*) for each experimental group/condition, given as a discrete number and unit of measurement
☐	☒	A statement on whether measurements were taken from distinct samples or whether the same sample was measured repeatedly
☐	☒	The statistical test(s) used AND whether they are one- or two-sided<br>*Only common tests should be described solely by name; describe more complex techniques in the Methods section.*
☐	☒	A description of all covariates tested
☐	☒	A description of any assumptions or corrections, such as tests of normality and adjustment for multiple comparisons
☐	☒	A full description of the statistical parameters including central tendency (e.g. means) or other basic estimates (e.g. regression coefficient) AND variation (e.g. standard deviation) or associated estimates of uncertainty (e.g. confidence intervals)
☐	☒	For null hypothesis testing, the test statistic (e.g. *F*, *t*, *r*) with confidence intervals, effect sizes, degrees of freedom and *P* value noted<br>*Give P values as exact values whenever suitable.*
☒	☐	For Bayesian analysis, information on the choice of priors and Markov chain Monte Carlo settings
☒	☐	For hierarchical and complex designs, identification of the appropriate level for tests and full reporting of outcomes
☒	☐	Estimates of effect sizes (e.g. Cohen's *d*, Pearson's *r*), indicating how they were calculated

Our web collection on statistics for biologists contains articles on many of the points above.

Software and code

Policy information about availability of computer code

Data collection	Microsoft Excel v16.41, Prism 8 and 9, GeneSys 1.6.4.0, Gen5 3.8.1.0, Bio-Rad CFX Manager 3.1, LAS X 3.7.4, TopSpin 3.5.7
Data analysis	CellRanger v3.0.1, Seurat v4, CytoTRACE in R v4.2.2, BiNGO v3.0.5 app in Cytoscape visualization platform v3.9.1, velocyto v0.17.1640, scVelo v0.2.573, and Dynamo v1.1.041 in Python v3, CellRank v1.5.1, CellRanger ARC v2.0, Signac v1.9.0, pheatmap v1.0.12, dittoSeq v1.6, hybrid v2.0, scDblFinder v1.12.0, DoubletFinder v2.0, and Monocle3 v3.21

For manuscripts utilizing custom algorithms or software that are central to the research but not yet described in published literature, software must be made available to editors and reviewers. We strongly encourage code deposition in a community repository (e.g. GitHub). See the Nature Portfolio guidelines for submitting code & software for further information.

Data

Policy information about availability of data

All manuscripts must include a data availability statement. This statement should provide the following information, where applicable:

- Accession codes, unique identifiers, or web links for publicly available datasets
- A description of any restrictions on data availability
- For clinical datasets or third party data, please ensure that the statement adheres to our policy

Single-cell-RNA-seq datasets generated for this study have been uploaded to the NCBI GEO repository with accession number GSE229901. The preprocessed TCGA-GBM data was downloaded from the following publicly available publication, doi:10.1038/ng.3781 (Barthel et. al, 2017). Additional source data are provided with this paper. The Neftel et al. (2019) 10X and Smart-seq2 data were downloaded from the following publicly accessible site: https://singlecell.broadinstitute.org/

single_cell/study/SCP393/single-cell-rna-seq-of- adult-and-pediatric-glioblastoma. Source data are provided with this paper. The Delgado et. al 10X data were downloaded from the Gene Expression Omnibus with accession number GSE187875. All source data are provided with this paper.

Research involving human participants, their data, or biological material

Policy information about studies with human participants or human data. See also policy information about sex, gender (identity/presentation), and sexual orientation and race, ethnicity and racism.

Reporting on sex and gender	The human embryonic stem cells (hESCs) obtained for this study include H1 male cells and H9 female cells that have been previously validated per the NIH hESC Registry. For this initial study, we began by using the H1 male cells and performed intracranial stereotactic injections in female athymic nude mice.
Reporting on race, ethnicity, or other socially relevant groupings	Race, ethnicity, or other socially relevant groupings were not specifically relevant to this study and included those characterizing available reagents and datasets needed.
Population characteristics	Datasets downloaded from the Barthel et. al publication and Neftel et. al publications included patients with a diagnosis of primary GBM. The dataset downloaded from the Delgado et. al publication were primary human cell cultures derived from the cortical germinal zone of three different specimens at stages of peak neurogenesis.
Recruitment	No participants were directly recruited for this study. Patient recruitment for datasets downloaded for this study are indicated in the respective publications.
Ethics oversight	All human stem cell research related to this study has been approved by the Institutional Review Board and Embryonic Stem Cell Research Oversight Committee (IRB ID: 201709124 and ESCRO# 17-005, Washington University School of Medicine). All participants donating tissue signed informed consent prior to tissue banking.

Note that full information on the approval of the study protocol must also be provided in the manuscript.

Field-specific reporting

Please select the one below that is the best fit for your research. If you are not sure, read the appropriate sections before making your selection.

[x] Life sciences [ ] Behavioural & social sciences [ ] Ecological, evolutionary & environmental sciences

For a reference copy of the document with all sections, see nature.com/documents/nr-reporting-summary-flat.pdf

Life sciences study design

All studies must disclose on these points even when the disclosure is negative.

Sample size	Sample size is based on effect sizes from prior publications. Unless otherwise stated, experiments were carried out three or more times in three biologically independent samples.
Data exclusions	TCGA-GBM data pre-analyzed for telomere maintenance-related genetic variants, TERT expression, and telomere length was downloaded. Samples were filtered to include only the GBM disease type, exclude samples where TERT expression was 0 or not detected, include samples with TP53 mutations annotated as binarized wildtype (0) or mutant (1), and exclude samples with ATRX, TERC, or DAXX mutations or samples with TERT fusions.
Replication	Experiments were performed in three or more independent biological replicates. Data were reproducible.
Randomization	Samples, cells, and mice used in all experiments were randomized.
Blinding	For all experiments, investigators were blinded as to group allocation during data collection and analysis. For all mouse experiments, experiments were double-blinded--in regard to condition upon cell injection into mice and in regard to endpoints of live bioluminescence and neurological deficit-free survival.

Reporting for specific materials, systems and methods

We require information from authors about some types of materials, experimental systems and methods used in many studies. Here, indicate whether each material, system or method listed is relevant to your study. If you are not sure if a list item applies to your research, read the appropriate section before selecting a response.

Materials & experimental systems

n/a	Involved in the study
☐	☒ Antibodies
☐	☒ Eukaryotic cell lines
☒	☐ Palaeontology and archaeology
☐	☒ Animals and other organisms
☒	☐ Clinical data
☒	☐ Dual use research of concern
☒	☐ Plants

Methods

n/a	Involved in the study
☒	☐ ChIP-seq
☒	☐ Flow cytometry
☐	☒ MRI-based neuroimaging

Antibodies

Antibodies used	Antibodies used for immunoblotting include PDGFRA (CST, #3174S), phospho-PDGFRA (Y754, Abcam #ab5460), phospho-ERK (T202/4, CST #9101S), phospho-AKT (S473, CST #4060S), TP53 (CST #9282), and β-ACTIN (Santa Cruz #sc-47778).<br>Antibodies used for Chromatin Immunoprecipitation include normal rabbit IgG (CST #2729S), normal mouse IgG (EMD Millipore #CS200621), anti-trimethyl-histone H3 Lys4 (Millipore #05-745R), anti-histone H3 monomethyl K4 (Abcam #ab8895), and anti-trimethyl-histone H3 Lys9 (Millipore #07-442).
Validation	Validation of antibodies used in this study are reported on the respective company catalogue pages.

Eukaryotic cell lines

Policy information about cell lines and Sex and Gender in Research

Cell line source(s)	Human embryonic stem cells used in this study include H1 male and H9 female cell lines that are publicly available.
Authentication	Human embryonic stem cells used in this study have been previously validated per the NIH hESC Registry.
Mycoplasma contamination	All lines were confirmed negative for mycoplasma using a sensitive, enzyme-based detection kit (Lonza MycoAlert PLUS Assay).
Commonly misidentified lines<br>(See ICLAC register)	No commonly misidentified lines were used in this study.

Animals and other research organisms

Policy information about studies involving animals; ARRIVE guidelines recommended for reporting animal research, and Sex and Gender in Research

Laboratory animals	6-8 week old female athymic nude mice ages (CrTac:NCr-Foxn1nu, Taconic Biosciences) were used for this study.<br>Mice were housed in static microisolator caging on 1/8 inch corncob bedding with ad libitum access to Lab Diet 5053 chow and autoclaved water. Temperature was maintained between 70° +/- 2° F, humidity within 30-70%, and 12:12 hour dark to light cycle. All mice were free of Pneumonia Virus of Mice (PVM), Reovirus 3 (REO3), Sendai virus, Mycoplasma pulmonis, Minute Virus of Mice (MVM), Theiler's Murine Encephalomyelitis Virus (GDVII), Lymphocytic Choriomeningitis Virus (LCMV), Polyoma Virus, Mouse Rotavirus (EDIM), Ectromelia Virus (Mousepox), Mouse Adenovirus, K Virus, Mouse Parvovirus (MPV), Cytomegalovirus, Mouse Hepatitis Virus (MHV), Clostridium piliforme,Streptococcus pneumonia, Bordetella bronchiseptica, Streptobacillus moniliformis, Corynebacterium kutcheri, Salmonella spp. Citrobacter rodentium, murine pinworms and fur mites. Mice were euthanized if they were unable to feed, unable to walk, developed a seizure disorder, or if body weight loss exceeded 20% of original body weight or 20% less than age and sex-matched normal controls. Additionally, if the tumor itself ulcerated through the skull or if there was evidence of obvious local infections, then animals were euthanized. All unforeseen health concerns were discussed with veterinary staff with regard to the need for euthanasia.
Wild animals	No wild animals were used in this study.
Reporting on sex	In this initial study, male human embryonic stem cells were implanted in female athymic nude mice. This design was intended to 'wash out' potential sex differences during tumor formation, and female cells in male mice will be tested in the future to corroborate findings.
Field-collected samples	No field collected samples were used in the study.
Ethics oversight	Our animal protocol (#21-0083) adheres to NIH and American Association for Laboratory Animal Science (AALAS) guidelines and has been approved by our Institutional Animal Care and Use Committee (IACUC).

Note that full information on the approval of the study protocol must also be provided in the manuscript.

Plants

Seed stocks	*Report on the source of all seed stocks or other plant material used. If applicable, state the seed stock centre and catalogue number. If plant specimens were collected from the field, describe the collection location, date and sampling procedures.*
Novel plant genotypes	*Describe the methods by which all novel plant genotypes were produced. This includes those generated by transgenic approaches, gene editing, chemical/radiation-based mutagenesis and hybridization. For transgenic lines, describe the transformation method, the number of independent lines analyzed and the generation upon which experiments were performed. For gene-edited lines, describe the editor used, the endogenous sequence targeted for editing, the targeting guide RNA sequence (if applicable) and how the editor was applied.*
Authentication	*Describe any authentication procedures for each seed stock used or novel genotype generated. Describe any experiments used to assess the effect of a mutation and, where applicable, how potential secondary effects (e.g. second site T-DNA insertions, mosiacism, off-target gene editing) were examined.*

Magnetic resonance imaging

Experimental design

Design type	Resting state tumor progression
Design specifications	Animals were anesthetized with 1-1.5% isoflurane in a conduction chamber and then transferred to animal scanner bed at 1-1.5% isofluran and 0.8 L/min oxygen flow rate through a nose cone to maintain anesthesia during the scanning procedure. Respiratory motion was monitored during the imaging procedures using a pressure sensitive pad. Animal body temperature was maintained at 37°C by a temperature controlled circulating water. Acquisition for each animal included 1 scan of 9 slices at 5 mm thickness with an average acquisition time of 1 min.
Behavioral performance measures	N/A

Acquisition

Imaging type(s)	Structural
Field strength	9.4T
Sequence & imaging parameters	Images were acquired using a 86 mm ID volume transmitter coil and a 4-channel mouse brain CryoProbe array receiver coil. Fat-suppressed T2-weighted TurboRARE images were acquired of the brain using the following parameters: TR = 2500 s, TE = 33 s, matrix size = 256 x 256, spatial resolution = 700 micron, rare factor = 9.
Area of acquisition	Whole brain

Diffusion MRI ☐ Used ☒ Not used

Preprocessing

Preprocessing software	Images were analyzed and tumor volumes extracted using the semi-automatic segmentation analysis software ClinicalVolumes (ClinicalVolumes, London, UK).
Normalization	N/A
Normalization template	N/A
Noise and artifact removal	Respiration for each animal was maintained between 70-100 breaths/min under isofluorane anesthesia and imaged with fat suppression.
Volume censoring	Only T2-hyperintense lesions within the anterior cerebral hemispheres were included for tumor volume determination.

Statistical modeling & inference

Model type and settings	Acquired 2D images were stacked into a 3D volume within the software. The MR images were segmented and pixels inside the brain were assigned to a color-coded material mask. The volume of the tumor was estimated by adding up the individual voxel volumes inside the tumor while taking partial volume effects on the boundaries of the surface into account. From this the material mask, a 3D surface model reconstruction of the tumor was generated, enclosing all the segmented voxels.
Effect(s) tested	Tumor growth was investigated by calculating tumor volumes for each longitudinal time point and then fitting an exponential growth model by nonlinear regression using GraphPad Prism 7 package (GraphPad, California). Value of P < 0.05 is considered statistically significant.

Specify type of analysis: ☒ Whole brain ☐ ROI-based ☐ Both

Statistic type for inference (See Eklund et al. 2016)	Voxel-wise
Correction	N/A

Models & analysis

n/a	Involved in the study
☒	☐ Functional and/or effective connectivity
☒	☐ Graph analysis
☒	☐ Multivariate modeling or predictive analysis
